# The impact of childhood varicella vaccination on the incidence of herpes zoster in the general population: modelling the effect of exogenous and endogenous varicella-zoster virus immunity boosting

**DOI:** 10.1186/s12879-019-3759-z

**Published:** 2019-02-06

**Authors:** Christophe Sauboin, Katsiaryna Holl, Paolo Bonanni, Anne A. Gershon, Bernd Benninghoff, Stephane Carryn, Margaret A. Burgess, Peter Wutzler

**Affiliations:** 1 GSK Vaccines, Value Evidence, Wavre, Belgium; 2 University of Florence, Health Sciences, Florence, Italy; 3 Columbia University, Pediatrics, New York, NY USA; 4 GSK Vaccines, Medical Affairs, Wavre, Belgium; 5 GSK, Vaccines R&D, Wavre, Belgium; 6 Paediatrics and Child Health, University of Sydney, Sydney, Australia; 7 Department of Experimental Virology, University Hospital Jena, Jena, Germany; 8 Present address: Bayer AG, Epidemiology, Medical Affairs and Pharmacovigilance, Berlin, Germany

**Keywords:** Boosting, Varicella, Herpes zoster, Vaccination, Viral infection

## Abstract

**Background:**

A controversy exists about the potential effect of childhood varicella vaccination on Herpes Zoster (HZ) incidence. Mathematical models projected temporary HZ incidence increase after vaccine introduction that was not confirmed by real-world evidence. These models assume that absence of contacts with infected children would prevent exogenous boosting of Varicella-Zoster-Virus (VZV) immunity and they do not include an endogenous VZV immunity-boosting mechanism following asymptomatic VZV reactivation. This study aims to explore the effect of various assumptions on exogenous and endogenous VZV immunity-boosting on HZ incidence in the general population after introduction of routine childhood varicella vaccination.

**Methods:**

An age-structured dynamic transmission model was adapted and fitted to the seroprevalence of varicella in France in absence of vaccination using the empirical contact matrix. A two-dose childhood varicella vaccination schedule was introduced at 12 and 18 months. Vaccine efficacy was assumed at 65%/95% (dose 1/dose 2), and coverage at 90%/80% (dose 1/dose 2). Exogenous boosting intensity was based on assumptions regarding HZ-immunity duration, age-dependent boosting effect, and HZ reactivation rates fitted to observed HZ incidence. Endogenous boosting was the same as pre-vaccination exogenous boosting but constant over time, whilst exogenous boosting depended on the force of infection. Five scenarios were tested with different weightings of exogenous (Exo) - endogenous (Endo) boosting: 100%Exo–0%Endo, 75%Exo–25%Endo, 50%Exo–50%Endo, 25%Exo–75%Endo, 0%Exo–100%Endo.

**Results:**

HZ incidence before varicella vaccination, all ages combined, was estimated at 3.96 per 1000 person-years; it decreased by 64% by year 80 post vaccine introduction, for all boosting assumptions. The 100%Exo-0%Endo boosting scenario, predicted an increase in HZ incidence for the first 21 years post vaccine introduction with a maximum increase of 3.7% (4.1/1000) at year 9. However, with 0%Exo-100%Endo boosting scenario an immediate HZ decline was projected. The maximum HZ incidence increases at 10, 3, and 2 years post vaccination were 1.8% (75%Exo-25%Endo), 0.8% (50%Exo-50%Endo) and 0.2% (25%Exo-75%Endo), respectively.

**Conclusions:**

Assuming modest levels of endogenous boosting, the increase in HZ incidence following childhood varicella vaccination was smaller and lasted for a shorter period compared with 100%Exo-0%Endo boosting assumption. Endogenous boosting mechanism could partly explain the divergence between previous HZ-incidence projections and real-world evidence.

**Electronic supplementary material:**

The online version of this article (10.1186/s12879-019-3759-z) contains supplementary material, which is available to authorized users.

## Background

Varicella (chickenpox) is a highly contagious infectious disease with a peak incidence among preschool and school-aged children. It is caused by the varicella-zoster virus (VZV) [1, 2]. After primary infection, VZV remains latent in neural ganglia until potential reactivation. Herpes zoster (HZ), also called shingles, is caused by the symptomatic reactivation of VZV. This reactivation is assumed to be a consequence of age-related decline of immunity in older adults or of a health condition that decreases the immune function such as for immunocompromised individuals [3–5].

Varicella is considered as a self-limiting disease which annually infects a large number of people, mostly children, almost equal to the size of the annual birth cohort in temperate regions. The disease can lead to serious complications in 3% of cases under 15 years of age and 6% of cases above 15 years of age in France, including secondary bacterial infections of skin and lungs, sepsis, aseptic meningitis, encephalitis, and Reye syndrome [3, 4, 6]. Severe VZV infections therefore impose a large burden in terms of public health and health care resource utilization, resulting in large economic and societal impact.

The disease burden and the viral transmission of VZV are reduced by the routine use of varicella vaccine in children. Several live attenuated varicella vaccines have been developed, with well-established efficacy and safety profiles [4, 7, 8]. In countries where varicella is an important public health burden, the World Health Organization recommends the introduction of varicella vaccination into the routine childhood immunization program [8]. A significant decline in varicella incidence has been observed in countries where varicella vaccination has been introduced [9–12]. Despite this body of evidence, in some countries, varicella vaccination is not implemented due to concerns that varicella disease would be shifting to older age groups for which complications occur more frequently, and that varicella vaccination may increase HZ incidence in the older population [13, 14]. These potential negative effects are not observed in epidemiological data after 2-dose vaccination in the United States where varicella incidence is shown to decrease in all age-groups [15] and no specific impact of varicella vaccination is observed in the older population (> 65 years of age) [16].

The potential negative effects of routine childhood varicella vaccination (RVV) on HZ incidence in older population originate from the “exogenous boosting theory”, which postulates that individuals susceptible to HZ coming into contact with VZV-infected children could maintain their cell-mediated immunity (CMI), thereby reducing the risk of reactivation and developing HZ [17–19]. The consequence of this theory is that reducing VZV circulation would result in reduction of contacts with infected children and then immunity boosting events for older people who have had varicella in the past. Therefore, these people would experience lower CMI allowing VZV to reactivate. In 2000, using disease transmission models, Brisson et al. applied this immunity-boosting mechanism and predicted an increase in HZ incidence in older people following the implementation of RVV [20]. In contrast to these model projections, several published studies based on epidemiological data from countries with RVV have not established an association between vaccination and an increase in HZ incidence [16, 21–33]. A possible reason for the disparity between the mathematical models and the epidemiological data may be due to the role of endogenous boosting, resulting from asymptomatic VZV-reactivation [17, 34], since the early model by Brisson et al. and later models, focused only on exogenous boosting [20, 35, 36].

The alternative hypothesis of endogenous boosting suggests that internal factors (e. g. stress) can cause asymptomatic VZV reactivation and boost CMI, thus preventing HZ [34, 37, 38]. Asymptomatic reactivation of VZV has been shown to occur also in immunocompromised and immunocompetent individuals [39, 40]. Although endogenous boosting is likely to occur [41], the extent of its role at the population level and possible interplay with exogenous boosting remains unknown [41, 42].

In this study, we aim to explore the impact of childhood varicella vaccination on HZ incidence through scenarios with different relative weighting of both exogenous and endogenous boosting mechanisms. This may be of importance when modelling the public health impact of RVV on HZ.

## Methods

**Fig. 1:**
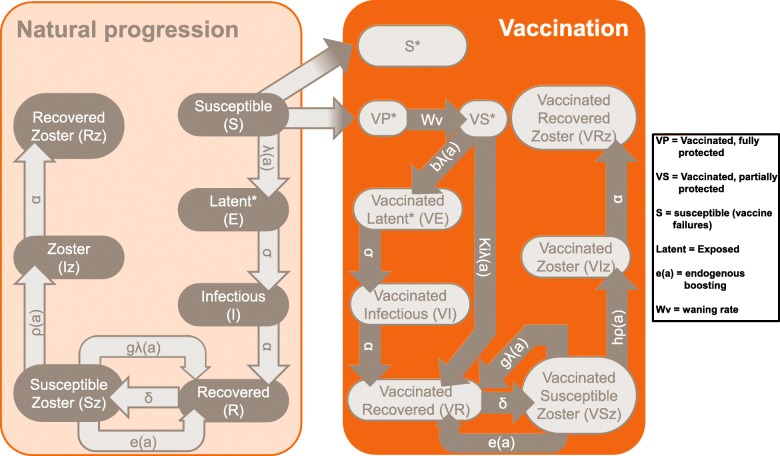
Model Structure [3, 4, 6]. Adapted from Ouwens et al. [43]. Addition of e(a) parameter to the model structure. Under public license CC BY-NC-ND 4.0.http://creativecommons.org/licenses/by-nc-nd/4.0/

The model we used for this study is an adaptation of the model developed by Ouwens et al. [43] which we modified by adding in an endogenous boosting effect. This population model is an age-structured dynamic transmission model (Fig. 1), developed in Matlab (version 2013b), with the same basic structure as the models by Brisson et al. [20, 35]. Our model is fitted to the age-specific VZV antibody seroprevalence from the French population in absence of vaccination and using an empirical contact matrix. The model reproduced varicella incidence with a plausible age-distribution. Age-specific VZV reactivation factors are then fitted to reproduce HZ incidence. Details of the dynamic model for France, the impact of the contact matrix, and exogenous boosting on varicella and zoster disease epidemiology have been previously reported by Ouwens et al. [43].

Briefly, the model structure (Fig. 1) combines varicella disease states as susceptible (S), i.e. not infected; latent or exposed (E), i.e. exposed to the virus but not showing symptoms; infected (I), which means showing symptoms and being infectious; and recovered (R) from the disease (model structure acronym: SEIR). The varicella part is followed by the zoster disease states as susceptible (Sz), infected (Iz), corresponding to symptomatic VZV reactivation, and recovered (Rz) which is a SIR-type model. Decreasing naturally-acquired VZV immunity with time (decreasing rate δ) causes recovered varicella patients to become susceptible to zoster, which means a transition from state R to state Sz, and0020exogenous boosting (gλ(a)) partially offsets this effect by triggering the opposite transition from state Sz to state R. The endogenous boosting (e(a)), which is a constant for each age a, is included as a transition from state Sz to state R in the natural progression part of the model, and from state VSz to state VR in the vaccination arm of the model. A main difference between endogenous and exogenous boosting effects is that the first one is considered constant over time but is varying over age whereas the second one varies over time as a function of the Force of Infection (FOI, λ), which denotes the rate at which susceptible individuals become infected per unit of time or alternatively measures VZV circulation in the population. Overall, in Fig. 1, the normal disease progression (before vaccination) arm is shown in black, and the disease progression in the vaccination arm in red.

The progression from state R to state Sz; then from state Sz to state Iz (as a result of VZV-reactivation) depends on three key parameters: (1) immunity duration against HZ after a varicella infection, (2) the risk of boosting event as a function of age when susceptible to zoster, and (3) VZV reactivation rates.

For the first parameter, the duration of immunity (1/δ) against HZ is unknown. Therefore, fixed values were assumed and tested in sensitivity analyses: 10 years in the base case and values of 2 and 20 years in the scenario analysis. Regarding the second parameter on the risk of exogenous boosting, the same values are used as in Brisson et al. [35] with a probability of boosting reducing with age from 75 to 50% after a contact with a varicella infected person (Table 1). Finally, the third parameter relates to the age-specific VZV reactivation rates (ρ(a)) that are calibrated to reproduce HZ incidence (reactivation rates are adjusted accordingly).

**Table 1:** Model parameters

Parameter	Description	Value	Source
Demographic parameters			
Birth rate	Fraction of annual birth cohort out of total French population	0.01295	INSEE, www.insee.fr [54]
Biological parameters			
σ	Latent period of varicella (average duration: 14 days)	26.07	Brisson et al. [20]
α	Infectious period of varicella (average duration: 7 days)	52.14	Brisson et al. [20]
δ	Waning natural immunity (average duration: 10 years)	0.1	Expert opinion
g * λ (a)	Exogenous boosting against zoster		Brisson et al. [35]
	< 50 years	75% * λ	
	50–64 years	71%* λ	
	> 65 years	50%* λ	
Vaccine parameters			
mmr1	Coverage of first dose of MMR French current coverage	90%	
mmr2	Coverage of second dose of MMR French current coverage	80%	
Dose1	Age at first vaccination (in months)	12	Assumption
Dose2	Age at second vaccination (in months)	18	Assumption
Introduction time	Number of years before maximum vaccination coverage is reached	3	Assumption
Tv	Varicella vaccine efficacy (% successfully vaccinated and temporarily protected)	65%	Prymula et al. [45]; NCT00226499
P	Varicella vaccine failures (%)	5%	Prymula et al. [45]; NCT00226499
1-Tv-P	Varicella vaccine-recipients partially protected (%)	30%	100%-Tv-P
Wv1	Waning rate for 1 dose of varicella vaccine (duration 17 years)	0.0588	Silverman et al. [55]
Wv2	Waning rate for 2 doses of varicella vaccine (lifelong protection)	1e^− 6^	Expert opinion
Ki * λ (a)	Rate of exogenous boosting	0.91 * λ (a)	Brisson et al. [20]
h	Relative VZV reactivation after varicella vaccination	0.167	Brisson et al. [20]
b * λ (a)	Rate of infection among vaccinated susceptibles	0.73 * λ (a)	Brisson et al. [20]
m	Relative infectiousness of infected vaccine-recipients versus non-vaccine-recipients	0.5	Brisson et al. [20]

Note: *MMR* measles, mumps and rubella, *MMVR* measles, mumps, rubella, and varicella, *INSEE* National Institute of Statistics and Economic Studies (Institut national de la statistique et des études économiques), *VZV* varicella zoster virus, *WHO* World Health Organization

Incidence was presented following the French age groups of preschool and school children, young adults, working adults, and older-age population: < 1 year, 1–4 years, 5–9 years, 10–14 years, 15–24 years, 25–44 years, 45–64 years, and ≥ 65 years. The lack of population-level data on endogenous boosting does not allow a full calibration of these parameters. Therefore, the constant age-dependent e(a) was assumed to be the same as the exogenous boosting before vaccination (pre-vaccination equilibrium). This level of boosting was derived by Brisson et al. from results of a trial on the live-attenuated HZ vaccine efficacy [35, 44].

Five different relative weights of endogenous and exogenous boosting effects were tested as different scenarios in the model (100%Exo-0%Endo, 75%Exo-25%Endo, 50-%Exo50%Endo, 25%Exo-75%Endo, and 0%Exo-100%Endo) so that the total force of boosting combining both exogenous and endogenous effects was assumed to be the same before vaccine introduction in all scenarios. In absence of data on the boosting effect, we set the total force of boosting to what was assumed by Brisson et al., [35] i.e. 75% for people ≤50 years, 71% for 51–69 years, 57% for 70–79 years and 32% for ≥80 years.

The model output was the evolution of HZ incidence over time after childhood varicella vaccine introduction in each scenario.

Tables 1 and 2 present the model input parameters and sensitivity analyses, respectively. Table 2 indicates the vaccination coverage for each dose, time for gradual implementation of varicella vaccination, vaccine efficacy waning assumptions and the type of age-structured contact matrix which is the same as in Ouwens et al. [43]. The table also include the resulting reactivation rates for HZ obtained after calibration given the assumptions on boosting rate and duration of immunity against HZ (10 years in the base case and 2 to 20 years in the sensitivity analysis). Ageing mechanism was included in the model. Each year, a cohort of newborns is introduced in the population and an age-specific mortality factor was applied while the rest of the population is moved to the older 1-year wide age-group. The mechanism assumed demographic equilibrium (the proportion of each age group remains constant over 100 years). A two-dose childhood varicella vaccination schedule was considered with doses given at the ages of 12 and 18 months.

**Table 2:** Base-case and sensitivity analyses

	Description	French coverage
Base case analysis	Vaccination coverage of MMR dose 1 and 2	Dose 1: 90%; Dose 2: 80%
	Time for replacement (MMR by MMRV)	3 years
	Catch-up program	No catch-up
	Exogenous/endogenous boosting	Included and relative weighting depends on scenario
	Contact matrix	Empirical
	Vaccine protection	Post-dose 1: 17 years; Post-dose 2: lifelong protection
Sensitivity analysis	Waning natural immunity (average duration 10 years – base case) changed to 2 and 20 years	bc: δ = 0.1 Low: δ = 0.05 High: δ = 0.5
	Reactivation rate of infectious zoster, by age group for δ = 0.1	
	0–4 years	0.028
	5–9 years	0.009
	10–14 years	0.0068
	15–24 years	0.0035
	25–44 years	0.0033
	45–64 years	0.008
	≥65+ years	0.016
	Reactivation rate of infectious zoster, by age group for δ = 0.5	
	0–4 years	0.00769
	5–9 years	0.00339
	10–14 years	0.00326
	15–24 years	0.00227
	25–44 years	0.00256
	45–64 years	0.00646
	≥65+ years	0.01387
	Reactivation rate of infectious zoster, by age group for δ = 0.05	
	0–4 years	0.05478
	5–9 years	0.01666
	10–14 years	0.01183
	15–24 years	0.00548
	25–44 years	0.00453
	45–64 years	0.01035
	≥65+ years	0.01991

δ, waning natural immunity (average duration 10 years), *bc* base case (scenario), *MMR* measles, mumps, and rubella, *MMRV* measles, mumps, rubella, and varicella

Vaccine efficacy was obtained from the clinical trials and assumed to be 65% for the first, and 95% for the second dose [45]. Vaccine coverages, of 90% for dose 1 and 80% for dose 2, were assumed with gradual scaling-up of varicella vaccination coverage over 3 years. No catch-up program was introduced into the model. The last two assumptions are different from Ouwens et al. [43] who have assumed 80% replacement of measles, mumps and rubella (MMR) by measles, mumps, rubella, and varicella (MMRV), and a 50% catch-up program.

## Results

**Fig. 2:**
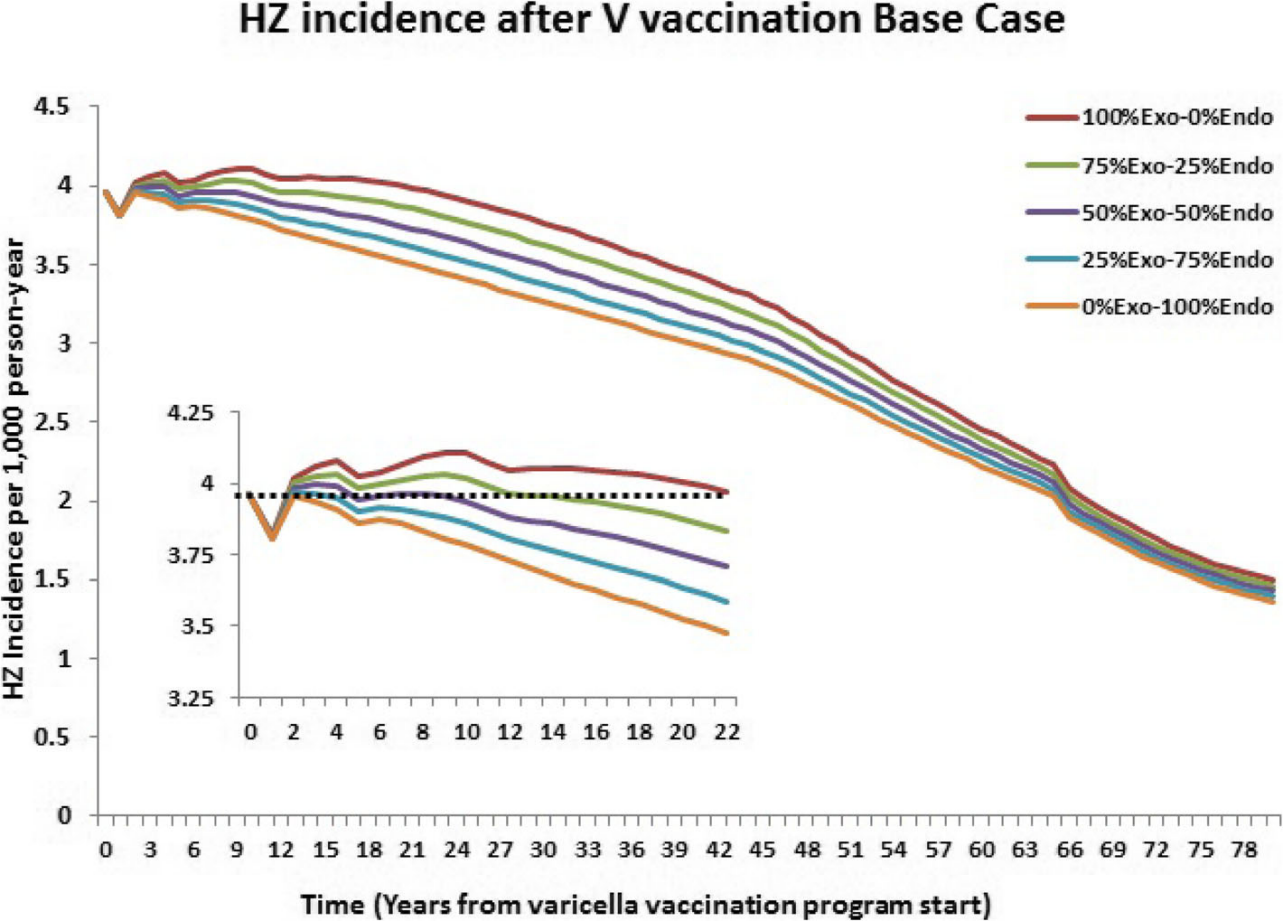
Trend in post-vaccination HZ incidence by scenario. HZ, herpes zoster; V, varicella

HZ incidence was well reproduced by the mathematical model for each age group and all boosting- effect assumptions. Before varicella vaccine introduction, the overall population HZ incidence was estimated at 3.96 per 1000 individuals, increasing with age (Table 3). Eighty years after varicella vaccine introduction, HZ incidence decreased by approximately 60% and was consistent across all Exo-Endo scenarios (Table 4). This decrease resulted from the lower risk of developing HZ in varicella vaccinees as they age. The 100% exogenous boosting effect and with 10 years of VZV immunity, however, resulted in a temporary initial increase (3.7%) in HZ incidence in the general population for the first 21 years after vaccine introduction (Fig. 2). In contrast, 100% endogenous boosting resulted in an immediate decrease in HZ after varicella vaccine introduction.

**Table 3:** Pre-vaccination HZ incidence by age group

All ages	< 5 y	5-9y	10-14y	15-24y	25-44y	45-64y	≥65y
3.96	1.03	1.69	2.33	1.87	1.87	2.12	9.09

*y* years of age

**Table 4:** Base-case scenario and sensitivity analysis results

HZ incidence parameters	Exogenous-Endogenous (%)				
	100–0	75–25	50–50	25–75	0–100
2-year immunity to HZ scenario					
Decrease in HZ by year 80 (%)	63.6	63.8	64.0	64.2	64.4
Number of years with HZ increase above pre-vaccine rate	3	3	2	1	0
Max HZ increase (%) above pre-vaccine rate	1.3	0.8	0.3	0.0	0.0
Year at max HZ increase	3	3	2	2	NA
10-year immunity to HZ scenario (base case)					
Decrease in HZ by year 80 (%)	62.1	63.1	64.0	64.8	65.6
Number of years with HZ increase above pre-vaccine rate	21	10	3	2	0
Max HZ increase (%) above pre-vaccine rate	3.7	1.8	0.8	0.2	0.0
Year at max HZ increase	9	4	3	2	NA
20-year immunity to HZ scenario					
Decrease in HZ by year 80 (%)	60.1	61.8	63.3	64.7	66.0
Number of years with HZ increase above pre-vaccine rate	33	23	9	3	1
Max HZ increase (%) above pre-vaccine rate	5.7	2.6	1.0	0.9	0.8
Year at max HZ increase	19	9	1	1	1

*HZ* herpes zoster, *NA* not available

For the other 3 scenarios (Exo-Endo 75–25%, Exo-Endo 50–50%, Exo-Endo 25–75%), higher proportions of endogenous boosting led to lower temporary increase in HZ incidence of 1.8, 0.8 and 0.2% and for shorter duration of increase of 10, 3, 2 years respectively (Table 4).

In addition, higher proportions of endogenous boosting lead to shorter time to maximum temporary increase in HZ incidence: 6 years for 25%Exo–75%Endo, 8 years for 50%Exo–50%Endo, 9 years for 75%Exo–25%Endo.

### Sensitivity analysis

The impact of introducing an endogenous boosting effect on HZ incidence in the general population was less important with the 2-year VZV immunity scenario, while a substantial difference was observed for 20 years of VZV immunity (Table 4).

Specifically, for 100% exogenous boosting, with the scenario of 2-year VZV immunity in comparison to the base-case scenario (10 years), the temporary initial increase in HZ incidence was projected to be smaller (1.3% versus 3.7%) and shorter (3 years versus 21 years). Thus, the estimated impact of increased endogenous boosting effect was smaller in the 2-year immunity scenario than in the base case.

When 20-year VZV immunity was assumed and with 100% exogenous boosting, the temporary initial increase in HZ incidence was projected to be higher (5.7% versus 3.7%) and to last longer (33 years versus 21 years) than in the base-case scenario. On the other hand, introducing 25% of endogenous boosting had a larger impact, with a lower maximum HZ incidence increase (− 3.1 percentage points, from 5.7 to 2.6%) and a shorter duration of HZ incidence compared to pre-vaccination levels (− 10 years from 33 to 23 years).

**Fig. 3:**
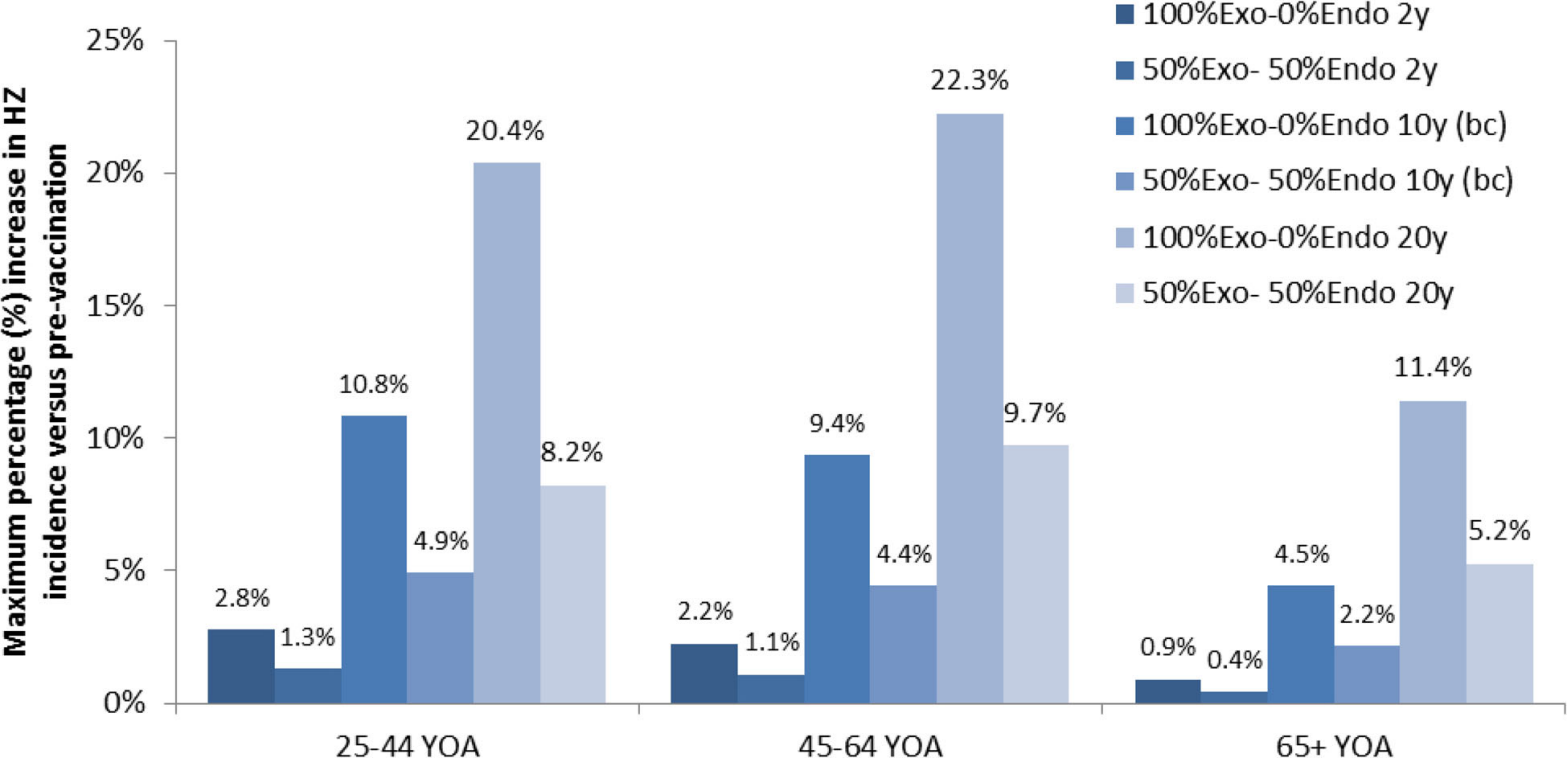
Maximum HZ increase, in full and partial exogenous boosting, by age groups and immunity scenarios. bc, base case (scenario); HZ, herpes zoster; y, years (of immunity).); YOA, years of age

The maximum estimated increases in HZ incidence compared to the pre-vaccination rate in the base- case scenario were in the group 25–44 years of age (Fig. 3), although the absolute increase in incidence was larger in the group 45–64 years of age (not shown on the figure). In the scenario with 20 years of HZ immunity, the maximum increase was projected in the 45–64 age group. When 50%Exo-50%Endo was considered, the maximum temporary initial increase was reduced by 53% in both the base case and in the 20-year HZ-immunity scenario, and by 51% in the 2-year HZ-immunity scenario.

Additional file 1 summarizes the content of the study in a form that could be shared with patients by healthcare professionals.

## Discussion

A small number of studies have attempted to model the effect of childhood varicella vaccination on the incidence of HZ in the population in the presence of endogenous boosting and its relative weight with exogenous boosting. Our analysis shows that even with a limited fraction of endogenous boosting, a substantial reduction occurs in the duration and magnitude of temporary initial HZ incidence increase after introduction of vaccination relative to pre-vaccination levels.

The current insight from the model means that relative weighting of exogenous and endogenous boosting effects may have an important and varied impact on VZV immunity and predicted HZ burden in the population.

Previous modelling studies projected that RVV would increase HZ incidence in the general population above pre-vaccination levels for about 20 to 50 years after the introduction of childhood vaccination under assumption of reduced exogenous boosting [20, 35, 36, 43, 46], and would decrease HZ incidence after 50 years of RVV. The individual-based dynamic transmission model of Ogunjimi et al. [46] predicted that the increase in HZ incidence would mainly affect people between 31 and 40 years of age and would decrease over time. Our model predicted a maximum relative increase for the 25–44-years age group and was therefore similar to Ogunjimi’s finding [46]. However, we found the absolute increase to be higher in the 45–64 years age group. When we compare with real-world data in the United States, despite some long-term increasing trend, epidemiological data have not shown an increase in HZ incidence related to the introduction of RVV in numerous countries [16, 21–33]. In a cohort study conducted in collaboration with the Centers for Disease Control and Prevention in 8017 patients with HZ, Kawai et al. [32] concluded that the more-than-4-times increase in HZ incidence over the past 60 years could not be attributed to the introduction of varicella vaccination. They examined the HZ incidence for the periods 1945–1960 and 1980–2007 and found that the incidence of HZ increased at the same rate before and after the introduction of varicella vaccination [32]. A Medicare study also found no association between the increase in HZ incidence and the introduction of varicella vaccination over 19 years (1992–2010) (relative risk of 0. 9998; 95% confidence interval, 0. 9997 to 1. 0022) [16]. At the June 2017 Advisory Committee on Immunization Practices meeting, discussions highlighted that none of the United States studies showing an increase in HZ incidence trends, showed evidence of association with varicella vaccination [33]. A recent systematic literature review of HZ risk reduction through exposure to varicella patients concluded that exogenous boosting exists, but may not be applicable to all situations, and that its magnitude is yet to be determined adequately in future epidemiological studies [34]. Real-world evidence rather suggests that factors other than exogenous, or even endogenous boosting, may play a role in HZ increase in the population, such as the use of immunosuppressive drugs [47]. After revising earlier mathematical models and incorporating recent epidemiological data, another study concluded that generalisations across different countries could not be made, and that the country-specific epidemiology of varicella affects the predicted impact of RVV on HZ [42]. In our modelling study, calibration was performed using only epidemiological data from France, which is a limitation considering that the initial level of exogenous boosting may vary from one country to another. However, some simulations done with data from another country show similar pattern on HZ incidence when introducing endogenous boosting (data not shown).

Using varicella and HZ data from the Netherlands, Marinelli et al. [48], compared the performance of fitting various sets of model parameters based on statistical criteria: Akaike information criterion (AIC) and Bayesian information criterion (BIC). Both criteria modulate the quality of the fit obtained by introducing a penalty on the number of parameters used to fit the data, the penalty being more important for BIC. Interestingly, introducing a constant endogenous boosting rate over age was the best fit for AIC, while excluding endogenous boosting was the best fit for BIC. It should be noted however that a single parameter was used to characterize exogenous boosting across all ages. This implies that the probability of immunity boosting after a contact with a varicella infectious individual is constant over age which is not consistent with previously-published VZV models. Large ranges of HZ-immunity durations have been assumed in the literature (up to 24 years of immunity). In our model, we assumed 10 years of immunity with a variation from 2 to 20 years in the sensitivity analyses. Results indicated a similar effect of a shorter 2-year duration of immunity than a longer 10-year immunity with some endogenous boosting. Assuming longer duration of HZ-immunity leads to a larger increase of HZ incidence after vaccination. In this case, the introduction of endogenous boosting further reduces that negative effect.

Moreover, complex interactions between exogenous and endogenous boosting mechanisms as well as other factors may exist, and the effect on HZ incidence may not be solely influenced by reactivation rates and exogenous boosting, as assumed in dynamic transmission models.

Introduction of HZ vaccination in older adults [49, 50], alongside the childhood varicella vaccination, could be a strategy to diminish the possible increase in HZ incidence in the general population. Previous studies that accounted for this approach in their model-based projections anticipated only a small effect of adult HZ vaccination on the predicted increase in HZ incidence in persons not vaccinated against varicella [36, 51]. However, this impact could increase substantially, depending on the effectiveness and duration of protection of the HZ vaccine as well as acceptance of the community to get a HZ vaccine [36].

Our study has limitations. The various assumptions (e.g. age at vaccination, vaccine coverage, duration of protection against HZ, and magnitude of exogenous and endogenous boosting), and the uncertainties around these assumptions can have a significant impact on model-based projections.

Some key parameters of the model like vaccination coverage, detection rate of HZ, demography and age-structured contact pattern are based on current knowledge and held constant over the analysis period. These are common hypotheses for population models in high-income countries. Also, due to the compartmental structure of the model, as opposed to individual-based models, heterogeneity in the immune-system status of individuals of the same age is not taken into account. Differences in HZ risk have been reported for different groups in the population e.g., higher risk in female, lower risk for black individuals [52]. Our model does not account for these differences within the population because we ignore if this reflects differences in boosting effect or another mechanism. Varicella disease seasonality was also not included in our model, first because the time-span of the model is long and is not likely to be affected by seasonality, second because it would increase the complexity of the calibration without enhancing the scientific question. HZ-related seasonality has not been established [53]. It is important to consider this modelling exercise as an exploratory analysis showing the potential role of various levels of endogenous boosting on the projected pattern of HZ incidence after varicella vaccination.

## Conclusions

In a VZV dynamic transmission model, assumptions on relative weighting of exogenous and endogenous boosting effects may have an important and varied impact on the predicted HZ burden following introduction of childhood varicella vaccination. The HZ burden in the general population is projected to decrease for all boosting scenarios, but a temporary increase in HZ precedes this effect when exogenous boosting alone is considered. This predicted increase in HZ is markedly reduced even when endogenous boosting is assumed to have a small weight. Endogenous boosting could therefore partly explain divergence between real-world evidence on HZ burden in countries utilizing childhood varicella vaccination and model projections based on exogenous-only boosting assumption. A safe and effective Varicella vaccine seems to offer the greatest promise controlling even long-term issues caused by this virus.

## Additional file

Additional file 1: Highlights / Patient Focus summarizes the content of the study in a form that could be shared with patients by healthcare professionals. (DOCX 13 kb)

## Abbreviations

AIC: Akaike information criterion
BIC: Bayesian information criterion
CMI: Cell-mediated immunity
Endo: Endogenous
Exo: Exogenous
FOI: Force of infection
HZ: Herpes zoster
INSEE: National Institute of Statistics and Economic Studies (Institut national de la statistique et des études économiques)
MMR: Measles, mumps and rubella
MMRV: Measles, mumps, rubella, and varicella
RVV: Routine (childhood) varicella vaccination
VZV: Varicella-zoster virus
WHO: World Health Organization
