# Supplementary material for: The impact of childhood varicella vaccination on the incidence of herpes zoster in the general population: modelling the effect of exogenous and endogenous varicella-zoster virus immunity boosting
Source: BMC Infect Dis. 2019 Feb 6;19:126. doi: 10.1186/s12879-019-3759-z (PMC6366068; doi:10.1186/s12879-019-3759-z)
Supplement: Supplementary file 1 — Highlights / Patient Focus summarizes the content of the study in a form that could be shared with patients by healthcare professionals. (DOCX 13 kb) [file 12879_2019_3759_MOESM1_ESM.docx]

***Highlights / Patient Focus***

- Exposure to circulating varicella-zoster virus (VZV) and asymptomatic endogenous reactivation are potential mechanisms to boost the immune response and to extend the protection against VZV reactivation as herpes zoster (HZ), these effects are referred to as exogenous and endogenous boosting effects, respectively.
- Mathematical models assuming a decrease in exogenous HZ immunity-boosting projected temporary HZ incidence increase after the introduction of childhood varicella vaccination. However, this population-level impact could not be established from observed data.
- A dynamic transmission model showed that different weightings of exogenous or endogenous boosting effects may substantially reduce the projected negative effect on HZ burden in the population.
- While both endogenous and exogenous immunity-boosting mechanisms have been shown to occur in individuals, this analysis suggests that the endogenous boosting effect could be one of the potential factors explaining the absence of negative effect in population-level observations suggested by previous models.
